# Magnetofection In Vivo by Nanomagnetic Carriers Systemically Administered into the Bloodstream

**DOI:** 10.3390/pharmaceutics13111927

**Published:** 2021-11-14

**Authors:** Artem A. Sizikov, Petr I. Nikitin, Maxim P. Nikitin

**Affiliations:** 1 Moscow Institute of Physics and Technology, 141701 Dolgoprudny, Russia; aasizikov88@gmail.com; 2 Prokhorov General Physics Institute of the Russian Academy of Sciences, 117942 Moscow, Russia; 3 National Research Nuclear University “MEPhI”, 115409 Moscow, Russia; 4 Department of Nanobiomedicine, Sirius University of Science and Technology, 354340 Sochi, Russia

**Keywords:** magnetofection in vivo, magnetic nanoparticles, iron oxide, gene delivery, gene vectors

## Abstract

Nanoparticle-based technologies are rapidly expanding into many areas of biomedicine and molecular science. The unique ability of magnetic nanoparticles to respond to the magnetic field makes them especially attractive for a number of in vivo applications including magnetofection. The magnetofection principle consists of the accumulation and retention of magnetic nanoparticles carrying nucleic acids in the area of magnetic field application. The method is highly promising as a clinically efficient tool for gene delivery in vivo. However, the data on in vivo magnetofection are often only descriptive or poorly studied, insufficiently systematized, and sometimes even contradictory. Therefore, the aim of the review was to systematize and analyze the data that influence the in vivo magnetofection processes after the systemic injection of magnetic nanostructures. The main emphasis is placed on the structure and coating of the nanomagnetic vectors. The present problems and future trends of the method development are also considered.

## 1. Introduction

**Figure 1:**
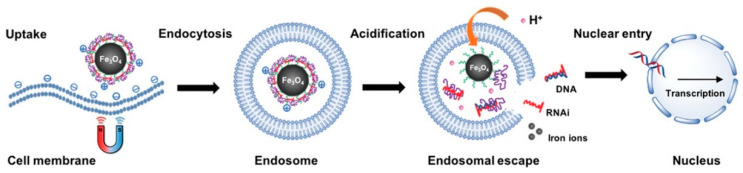
Magnetofection using magnetic nanoparticles. The plasmid DNA is associated with magnetic nanoparticles, which are directed, attracted, and concentrated on the surface of cell membranes, where the endocytosis process brings the nanoparticles into the cell. Adapted with permission from [43].

The development of nanosystems that effectively deliver genes into a cell in vivo using magnetofection is a complex and urgent task. A solution to this problem will lead to a significant progress in the creation of drug formulations for gene therapy. In such kind of therapy, nucleic acid molecules delivered into a cell can be used for over-expression of a desired protein, for gene knock down effects, for bypassing or even reparation of genetic mutations, or for activation of the innate immune system [1,2]. There are two main factors limiting biomedical applications of the technology. The first one is not fully understood the process of nucleic acids uptake, their intracellular interactions, intracellular trafficking and regulation of nucleic acid action inside cells at the molecular level. The second one is the direct delivery of nucleic acids to target cells [1,3]. Therefore, choice of the reliable and efficient delivery vectors is very important. When we talk about non-viral vectors, magnetic nanoparticles (MNPs) are often meant. They are considered to be in the center of nanotechnology-based structures for nanomedicine [4,5]. Due to their ability to respond to the magnetic field, MNPs have become very attractive for different theranostic applications [6,7,8]. When using MNPs, it becomes possible to carry out the magnetically controlled accumulation and release of these particles [9,10,11], execute the particle tracking by magnetic resonance imaging (MRI) [12,13,14], imaging of tumors [15,16], precise and quantitative monitoring in vitro [17,18,19] and in vivo [20,21], by the magnetic particle quantification (MPQ) technique in an extraordinarily wide linear dynamic range (up 7 orders of magnitude). MNPs themselves can be used for tumor therapy [22,23], for targeted drug delivery to a selected part of the body [22,23,24], or for the magnetic separation of cells [22], as well as for magnetofection [1,25,26]. Magnetofection is defined as method for nucleic acid delivery under the influence of a magnetic field acting on nucleic acid vectors that are associated with magnetic nanoparticles (see scheme in Figure 1) [27,28]. After reaching the cell surface, magnetic nanosystems (usually with polyethylenimine and DNA) are internalized into intracellular vesicles called endosomes as a result of endocytosis. Moreover, for the functional delivery of nucleic acids, so-called endosome escape is required. Otherwise, the magnetic nanoconstructions will be destroyed by the cellular enzyme system. It is believed that PEI–DNA complexes escape from the endosomes due to the so-called proton sponge effect [29]. Magnetic lipoplexes behave in a similar manner [30]. As in the case of nonmagnetic PEI polyplexes [31], a three-step behavior is observed. At the first stage, the magnetic lipoplexes attach to the cell surface and slowly penetrate the cell membrane. Most lipoplexes are internalized through endocytosis during this phase. The second stage is characterized by abnormal and limited diffusion within cells. The third stage is active transport along microtubules inside the cell.

Compared to the commonly used lipid or polymer-based transfection vectors, magnetofection has several advantages, such as higher efficiency, shorter delivery time, a possibility of very local delivery [32,33,34]. All the above-mentioned advantages of the method are especially relevant when performing magnetofection in vivo. There are several reviews in the literature devoted to various aspects related to in vitro [1,5,27,35,36,37,38,39,40,41] and in vivo [42] magnetofection. The latter work considers in vivo delivery of genes using magnetic carriers injected locally to targeted tumors or tissues [42]. In the current work, we provide an overview of the published results on the systemic injection of complex magnetic nanostructures into living organisms. The delivery of drugs/genetic information to the close vicinity of the site of action (tumor, target organ, etc.) is considered to be local. The administration into the circulatory system so that the entire body is affected is regarded as systemic. The main focus is on the magnetic vector structure. We discuss in detail different types of coating of various magnetic particles, give several examples of “unusual” magnetic carriers. Special attention is given to possible ways of development of the existing technology, challenges and prospects of the method.

## 2. Applications of Nanoscale Carriers for Magnetofection In Vivo

This review is a logical continuation of our previous work [42], where examples of in vivo magnetofection were described in detail for the case of administrating the magnetic vectors directly into a targeted tumor or tissue. Local injections actually allow to avoid such negative effects as undesired interactions with blood components and rapid elimination from the circulation by the reticuloendothelial system (RES). The aim of the present review is to collect and analyze data that influence the processes of magnetofection in vivo after systemic injections of the nucleic acid-bearing magnetic nanoparticles. We have summarized the related results available in various publications on the topic in Table 1.

**Figure 2:**
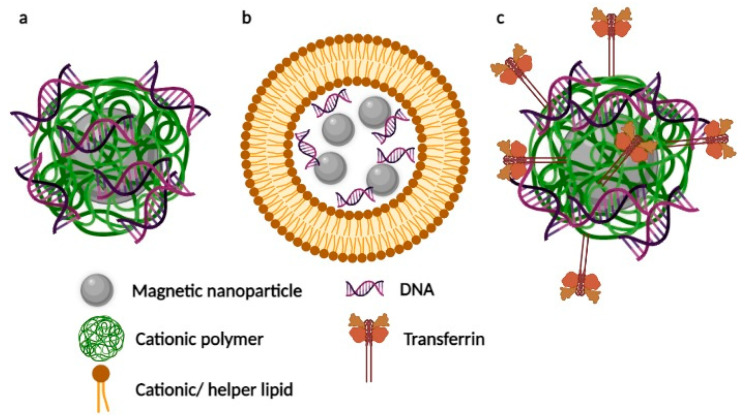
Schematic examples of different magnetic nanocarriers for magnetofection in vivo: (**a**) magnetic polyplex, (**b**) magnetic liposome, (**c**) magnetic polyplex modified with transferrin. Created with BioRender.com (accessed on 7 November 2021).

The efficiency of the in vivo application of one more magnetic nanocarriers critically depends on the charge and type of the magnetic core coating. Therefore, in this section, we have divided the data into three subsections: magnetic polyplexes (magnetic nanoparticles coated with cationic polymers, Figure 2a), magnetic liposomes (lipid-coated magnetic nanoparticles, Figure 2b), and magnetic nanosystems with an additional active targeting modality (along with the response to the magnetic field, Figure 2c). Furthermore, possible ways of the method evolution are discussed in detail.

### 2.1. Magnetic Nanoparticles Coated with Cationic Polymers

After the development of a method to synthesize a low aggregated magnetic polyethyleneimine/DNA nanostructures (MPD) and testing these particles when injected locally into a tumor [60], the same authors continued their research on the efficient transfection activity in a serum-containing medium and MPD application in vivo after intravenous injections [47]. The magnetic vector synthesis is described in [60]. Shortly, MNPs were synthesized by two different methods in aqueous [61] and organic media [62] with subsequent modification by ligand exchange or by silane-coupling agents to prepare a negatively charged coating. For this purpose, *N*-(trimethoxysilylpropyl) ethylenediaminetriacetic acid was used. As a result, the luciferase gene expression in tumors substantially differed for the traditional transfection and magnetofection groups. The luciferase activity effected by the transfection with MPD nanostructures subjected to a magnetic field was about five times stronger compared to that obtained without field application or by the control standard PEI-DNA complexes. It is also worth noting that a large portion of the particles in all cases settled in the animal’s lungs.

**Figure 3:**
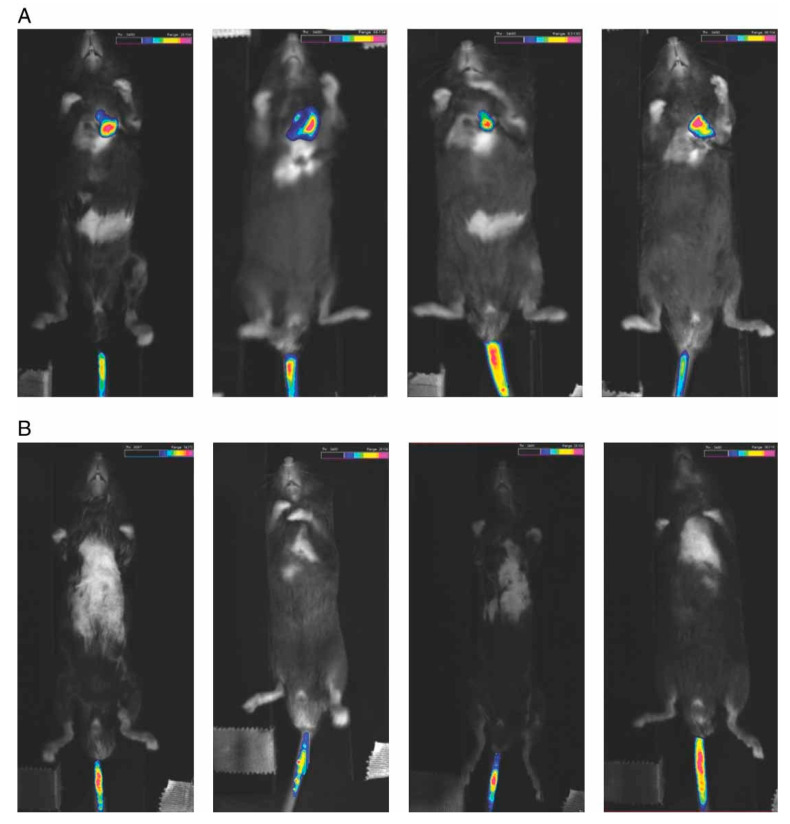
Non-invasive in vivo trafficking of MNB/PEI/DNA nanostructures 2 h after systemic administration. The fluorescent image (pseudocolor) was overlaid on the photographic image. The intensity of fluorescent signal from MNB/Oregon green 488 labeled-PEI/DNA was stronger in the left chest in group with magnet (**A**) compared to the group without magnet (**B**). Adapted with permission from [48].

In contrast, an even higher luciferase activity was recorded, when commercial magnetic nanoparticles were used, namely MNBs (MiltenyiBiotec, Auburn, CA, USA) with an average size of 200 nm [48]. The magnetic polyplex was prepared as follows: biotinylated PEI/DNA complexes were conjugated to MNBs via a sulfo-NHS LC-biotin linker. Two hours after systemic administration of the labeled MNB/Oregon green 488-PEI/pREP4/Luc nanostructures, their accumulation was assessed in vivo by a bioimaging system that detected fluorescence. When using a magnet, the intensity was about 18 times higher in the left side of the animal’s chest than without the magnetic field (Figure 3), indicating that the magnet effectively attracted the nanostructures to the left chest (heart and left lung).

Additionally, the magnetic field application caused strong therapeutic gene expression in the left lung and heart. Unfortunately, there was no quantitative assessment as in another publication with similar particles [57], where the level of luciferase gene expression in the heart increased more than three orders of magnitude (!) compared with the control mice without implantation of an epicardial magnet. Another example of work using magnetic vectors based on commercial nanoparticles is discussed in [49]. To investigate whether magnetofection could be a feasible strategy for directing antisense ODN to one specific vascular site after an intra-arterial injection in vivo, the authors infused mice with Cy3-labeled antisense ODN complexed to PolyMAG magnetic particles through a femoral catheter. As a result, solely all large and small arterioles of the cremaster muscle exposed to the magnetic field after the injection of the ODN-MP mixture showed a high level of fluorescence. That was not the case in the vessels of the cremaster muscle of the contralateral testis in the same animals. In these control vessels, similar to the control animal, which was injected with a magnetic vector without applying a magnetic field, only a few large arterioles and none of the small arterioles showed fluorescence, which qualitatively indicated that magnetofection is a working method for active targeting.

The work of [53] aimed at estimating the targeting capacity in vivo under the influence of a magnetic field of angiopoietin-2 small-interfering RNA (Ang-2 siRNA) plasmid/chitosan-coated magnetic nanoparticles in a model of malignant melanoma (MM) in nude mice. The method of obtaining chitosan magnetic nanoparticles was quite interesting. At the first stage, magnetic Fe_3_O_4_ nanoparticles were dispersed in a chitosan solution (Zhejiang Hisun Chemical Co., Ltd., Taizhou, China) under ultrasound agitation. Subsequently, the mixture was added to a mixed phase solvent of liquid paraffin and petroleum ether supplemented with Span-80. The solution was sufficiently emulsified and agitated, then glutaraldehyde solution was slowly added dropwise. Then, the solution was incubated at 40 °C in a water bath for 30 min followed by adjustment pH to 9.0 with NaOH solution. The resulting solution was heated to 60 °C. After standing for 1 h, the precipitate was produced. Then, successive thorough washings with anhydrous ether, acetone, anhydrous ethanol, and distilled water produced the chitosan magnetic nanoparticles. As a result, it was shown, at a qualitative level, that the targeting group (particles + magnetic field) exhibited aggregation of numerous particles on the capsule of tumor tissues and inside blood vessels and staining with Prussian blue was strictly positive.

Another example of using custom-made magnetic particles is given in [51]. It was proposed to use chondroitin sulfate-polyethylenimine copolymer (CP)-coated superparamagnetic iron oxide nanoparticles as an efficient magneto-gene carrier for microRNA-encoding plasmid DNA delivery. The magnetofection gene carrier was prepared by complexation through electrostatic interactions between CP and self-made [63] poly(acrylic acid)-bound iron oxide nanoparticles (SPIONs) (named CPIO). To evaluate the in vivo magneto-induced uptake of the magnetoplex, a biodistribution of CPIO/Cy5-DNA was studied in nude mice with U87-xenografted tumors on the right and left hind leg regions. As a result, the enhanced permeability and retention (EPR) effect [64] played a greater role in biodistribution than the magnetic field (after 48 h, both tumors glowed, the one above, where the magnet was located, glowed slightly stronger).

Magnetic mesoporous silica nanoparticles (M-MSN) are an unusual example of a magnetic vector for in vivo magnetofection. The research of [54] aimed at creating M-MSNs that differed in shape followed by a comparative study of their efficiency in suicide gene therapy of hepatocellular carcinoma (HCC). As a model, the thymidine kinase/ganciclovir of herpes simplex virus (HSV-TK/GCV) gene therapy system was used. Carboxyl-functionalized M-MSNs were first loaded with GCV. Then, PEG-g-PLL was introduced to ensure a positive surface charge of the resulting nanostructure for electrostatic absorption of the TK plasmid. At the first stage, nanosized magnetite coated with polyacrylic acid was obtained [65]. Then, spherical (S-M-MSNs) and rod-shaped (R-M-MSNs) mesoporous silica nanoparticles were synthesized by a simple sol-gel method using the obtained Fe_3_O_4_ NPs and cetyltrimethylammonium bromide (CTAB) as a template [66,67]. After that, carboxylate-modified M-MSNs were formed via reactions of M-MSNs with ammonium persulfate (APS) and further with succinic anhydride [68,69]. Finally, PEG-g-PLL and M-MSNs–COOH were covalently conjugated using a modified EDC/NHS reaction according to [70,71]. To evaluate the therapy results, the authors proposed a comparison of the sizes of the respective tumors. Compared to the control group, which was given only saline, tumor growth inhibition was observed in all other cases. In addition, the group that was treated with the magnetic field showed a significant reduction in the relative tumor volume and weight compared to the non-magnetic group. Moreover, if both types of the field (constant and variable) were used, then the tumor was less than under a single field type, while the latter, in turn, was less than without any field.

### 2.2. Lipid-Coated Magnetic Nanostructures

The use of magnetic lipoplexes via magnetic field-assisted systemic delivery appeared to be a much more popular method compared to the local delivery [42]. A team of authors led by Aiqiang Dong believes that liposomal magnetofection works best when administered systemically and that the magnetic field potentiates gene transfection to concentrate magnetic lipoplexes onto target cells [44,45,46]. In all three papers, the magnetic nanosystems had the following composition: pGFPshIGF-1Rs/combiMAG/lp2000. In [44], it was shown that the silencing efficiency of shRNAs delivered by the liposomal magnetofection after a pGFPshIGF-1R injection reached 43.4 ± 5.7%, 56.3 ± 9.6%, and 72.2 ± 6.8% at 24, 48 and 72 h, respectively, and reached an average of 43.8 ± 5.3% by lipofection. The biological distribution and target tumor suppression after magnetofection were also studied along with the potential toxicity of the method via combiMAG-carrying plasmids expressing green fluorescent protein (GFP) and short hairpin RNAs (shRNAs) targeting IGF-1R (pGFPshIGF-1Rs) in tumor-bearing mice [45]. In that work, the accumulation and delivery of pGFPshIGF-1R into tumors using magnetic nanoparticles and a magnetic field contributed to a significant decrease in tumor growth compared to the control, the suppression rate was 36% on day 30 after treatment. The same magnetic lipoplex can be used for gene therapy after heart failure [46]. The results showed that the silencing efficiency of shRNAs delivered by liposomal magnetofection reached 72.2 ± 6.8, 80.7 ± 9.6 and 84.5 ± 5.6%, at 24, 48 and 72 h, respectively, after pGFPshIGF1R injection.

Namiki Y. et al. [10] described the development of a new nanoparticle, which consisted of an oleic acid-coated magnetic nanocrystal core and a cationic lipid shell (DOTAP (*N*-[1-(2,3-dioleoyloxy) propyl]-*N*,*N*,*N*-trimethylammonium chloride) and DOPE (dioleoylphosphatidylethanolamine) (1:1)). The lipid mixture dissolved in chloroform was mixed with chloroform-based magnetic fluid. After the addition of distilled water, the homogeneous mixture was evaporated, sonicated, and purified, finally forming LipoMag. LipoMag/siRNA^EGFR#4-mU^ (siRNA to efficiently knock down the EGFR mRNA in tumor vessels) treatment under a magnetic field exhibited a ≈50% reduction in the tumor volume compared with the control group on day 28th after the treatment initiation.

**Figure 4:**
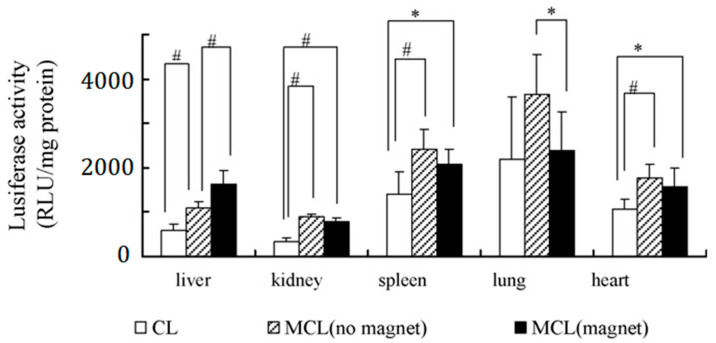
Effect of external magnetic field on the transfection activity of MCLs/pDNA nanoformulations in vivo. The concentration of MAG-T in the MCLs was 1.5 mg/mL and the weight ratio between MCLs and pDNA was 8.0. Luciferase activity was determined 24 h post-injection in the liver, kidney, spleen, lung, and heart, respectively. Each value represented mean ± S.D. (*n* = 6). * *p* < 0.05, # *p* < 0.01. Adapted with permission from [50].

It is known that liposomes composed of DC-Chol(3β-[*N*-(*N*’,*N*’-dimethylaminoethane)-carbamoyl] cholesterol) and DOPE (Dioleoylphosphatidylethanolamine) have been classified as one of the most efficient vectors for transfection of pDNA into cells and in clinical trials [72,73,74]. In the work [50], DC-Chol and DOPE (1:1 molar ratio) were chosen as the liposome composition, MAG-T (aqueous dispersion of magnetite Fe_2_O_3_; tartaric acid matrix; mean diameter of 20 nm) was used as the core, and the MCLs were prepared using reverse-phase evaporation. Since the target organ was the liver, the observed luciferase activity 24 h after injection was only one when using MCLs/pDNA nanoformulations under the influence of a magnetic field and 1.5-fold higher compared to the same particles without the field (Figure 4).

Another interesting example employing magnetic liposomes is presented in [58]. Here, the delivery system was based on thermosensitive cationic liposomes, which were prepared with a thermosensitive cationic formulation of 1,2-dipalmitoyl-sn-glycero-3-phosphocholine (DPPC), 3b-[*N*-(*N*’,*N*’-dimethylaminoethane)-carbamoyl]cholesterol (DC-Cholesterol), dimethyldioctadecylammonium bromide (DOAB) and cholesterol at a molar ratio of 80:5:5:10 (TSCL liposomes). To prepare the magnetic liposomes (TSMCL), a magnetic fluid Fe_3_O_4_ was used as the core, which was co-encapsulated with ammonium sulfate buffer into the liposomes. The authors of the study [58] designed TSMCL-DOX-shSATB1 as a combined magnetic drug targeting and a magnetofection system to improve the efficiency of simultaneous delivery of DOX and SATB1 shRNAs. Co-delivery of DOX and the shSATB1 vector in an in vivo mouse xenograft model under the influence of a magnetic field resulted in weaker tumor growth compared to control mice.

After studying the behavior of micromagnetobubbles (MMB) through a local injection [75], the same authors continued their research trying systemic administration [59]. The endothelium of the treated dorsal skin clearly showed expression of the dsRed protein 48–72 h after treatment. That expression could not be observed in the vessel wall of mice treated with the plasmid-loaded MMB, where no external magnetic field and ultrasound had been applied, or in the equally treated mice, where only a magnetic field was applied but no ultrasound. Interestingly, although mice treated with only ultrasound but without a magnetic field showed low rates of transfection into the vascular wall in vivo, this effect was more than 60-fold lower than that observed when a magnetic field was added to retain the MMB at the vascular wall.

### 2.3. Magnetic Nanosystems Possessing an Additional Active Targeting Modality

**Figure 5:**
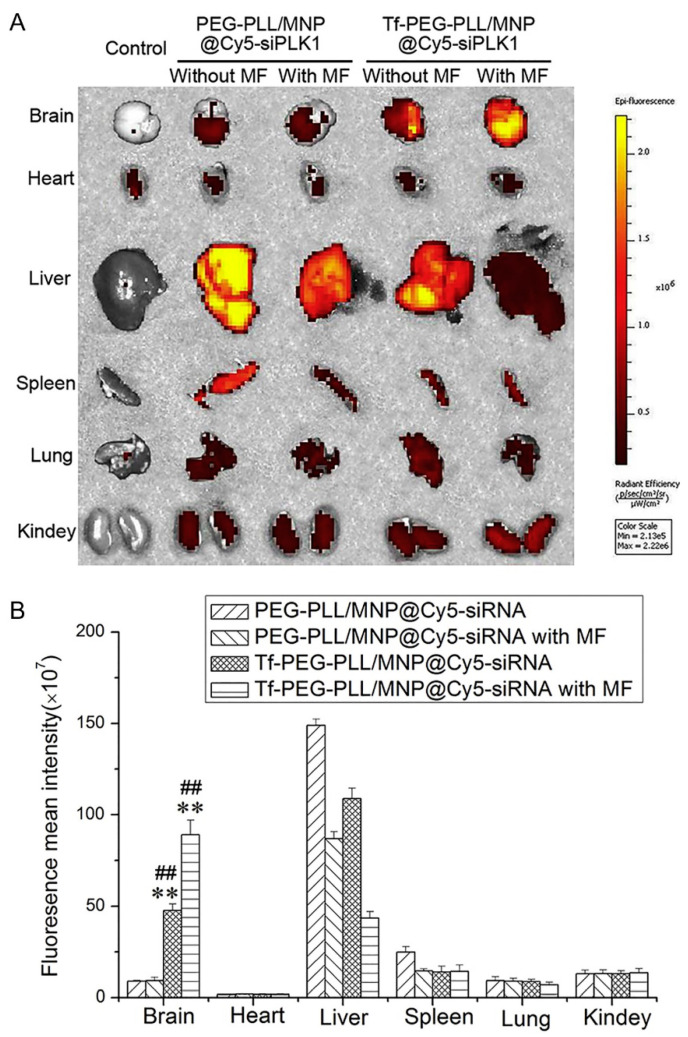
(**A**) Biodistribution of Cy5-siPLK1 in mice organs at 12 h after the administration of Tf-PEG-PLL/MNP@siPLK1 and PEG-PLL/MNP@siPLK1 by tail vein injection. MF presents the application of magnetic field. (**B**) The statistic results of panel A. ** *p* < 0.01, vs PEG-PLL/MNP@siPLK1 without MF; ## *p* < 0.01, vs PEG-PLL/MNP@siPLK1 with MF. Data are expressed as the mean ± SD (*n* = 3). Adapted with permission from [52].

In order to enhance the penetration of small interference RNA against the polo-like kinase I (siPLK1) across the blood–brain barrier to treat glioblastoma (GBM), magnetic nanoparticles (Tf-PEG-PLL/MNP@siPLK1) modified with transferrin (Tf) were prepared [52] and two types of active targeting were applied. Transferrins are iron-binding blood plasma glycoproteins that control the level of free iron (Fe) in biological fluids [76]. MNPs (Fe_3_O_4_) were prepared by alkaline co-precipitation [77] with subsequent linking with Tf-PEG-PLL or PEG-PLL. When evaluating in vivo anti-GBM activity, it was found that the tumor inhibition rate raised with increasing the dosage of the magnetic nanocarrier with trans-peptide (no-field conditions were not considered). The biodistributions of another self-luminous siRNA with and without application of the magnetic field were also evaluated (Figure 5).

The fluorescence intensity was significantly higher in the brain upon administration of Tf-PEG-PLL/MNP@Cy5-siPLK1 compared to PEG-PLL/MNP@Cy5-siPLK1 without transferrin. Moreover, it turned out that the magnetic field significantly increased the accumulation of Cy5-siPLK1 in the brain tissues. That, in turn, might contribute to the high activity of Tf-PEG-PLL/MNP @ siPLK1 in vivo against GBM.

The authors of Ref. [55] developed a nanovector with double targeting properties for the efficient delivery of a tumor suppressor gene RASSF1A specifically into hepatocellular carcinoma (HCC) cells by preparing galactosylated-carboxymethyl chitosan-magnetic iron oxide nanoparticles (Gal-CMCS-Fe_3_O_4_-NPs). It is known that galactose (Gal)-modified magnetic nanoparticles can be discerned specifically by the asialoglycoprotein receptor expressed on the surface of HCC cells and specifically recognized by HCC cells [78]. Fe_3_O_4_ NPs themselves were obtained in the aforementioned research by alkaline co-precipitation [77], then coated with chitosan [79] followed by the addition of lactose and sodium cyanoborohydride to obtain Gal-CMCS-Fe_3_O_4_ NPs. The authors studied the biodistribution of the particles loaded with pcDNA6.2mir-EGFP using fluorescent microscopy to visualize GFP expression. They noted green fluorescence in liver and tumor tissues. The average efficiency of pcDNA6.2mir-EGFP transfection in liver tissue was 32.6%. In addition, the average transfection efficiency in tumor tissue was approximately 40.8% and 29.7% when using an external magnetic field and without it, respectively. No overt fluorescence was observed in sections of kidney, spleen, heart, or lung tissues. As for delivery of the RASSF1A gene for HCC treatment, the best results were achieved when a combination of the magnetic vector, external magnetic field, and intra-abdominal administration of mitomycin (MMC—chemotherapy drug) were used for treatment.

The authors of [56] considered magnetic vectors, in which the core consisted of iron oxide modified by galactose (Gal) and polyethylenimine (PEI). The latter acted as shells providing targeted delivery of therapeutic siRNA to the liver cancer. Carboxylate-capped Fe_3_O_4_ was initially synthesized via the modified oxidative co-precipitation method [80]. Subsequently, PEI was further attached to the surface of Fe_3_O_4_–COOH via 1-ethyl-3-(3-dimethylaminopropyl)carbodiimide (EDC) followed by the addition of Gal-PEG-NH2. The biodistribution study of the Gal-PEI-SPIO/Cy5-siRNA particles showed rapid accumulation of Cy5-siRNA in the liver and tumor within 8 h. The fluorescence was much more pronounced compared to the case of nano-encapsulated Cy5-siRNA. Fluorescence from Cy5-siRNA was observed within 24 h and decayed significantly over time. With Gal-PEI-SPIO nanoparticles coated with Cy5-miRNA, the fluorescence intensity began gradually decreasing only after 8 h since administration. After 24 h, it was still observable and stronger than that in the control group (RNA only). It was found that the use of a magnetic field did not play a significant role in the inhibition of tumor growth. In general, tumors treated with Gal-PEI-SPIO/si-c-Met showed an obvious decrease in volume. c-Met is a hepatocyte growth factor (HGF) receptor that plays an important role in the proliferation, motility, differentiation, and angiogenesis of HCC [81]. As a result, both groups (with and without magnetic field), in which treatment was used with particles with siRNA, which silenced the expression of c-Met, demonstrated a significant (and almost the same) reduction in tumor volume compared to the control group.

## 3. Conclusions and Outlook

In this review, we have summarized the results of publications devoted to in vivo magnetofection for the case of systemic injection of magnetic nanocarriers and tried to figure out how it works depending on the structure of these magnetic vectors. Judging from the above-cited articles, we can say that the method proved to be a good tool for delivering genetic material (pDNA, siRNA, or antisense ODN, see Table 1) to tumors and tissues via magnetic nanoparticles with various types of coating. Due to the use of a magnetic field, a rather high delivery targeting is achieved at a low loading dose. All this moves magnetofection one step closer to practical applications in gene therapy.

Indeed, it has mentioned in a number of articles that the use of a magnetic field leads to higher gene expression on qualitative [45,49,51] and quantitative [47,48,50,52,56,57] levels. There is also numerical data on a significant decrease in tumor growth compared to control groups [10,45,52,55,56,58], as well as data on silencing efficiency [44,46], which indicate the positive effect of the magnetic field on the transfection process. A logical step would be to compare the results that scientific groups, which study in vivo magnetofection, obtained with the systemic and local routes of injection of magnetic nanostructures based on the same magnetic carriers. This is what the authors of [47,60] carried out. They declared in [47] that transfection by nucleic acid-bearing magnetic nanosystems under a magnetic field produced the highest level of luciferase activity, which was approximately 5-fold higher than that of the magnetic systems without field (when administered locally, the difference was 15-fold [60]). There is also a similar example from a group that has studied the transfection properties of micromagnetobubbles in vivo (local [75] and systemic [59] injections, respectively). Unfortunately, the results of these works could not be directly compared. There are no more examples of such comparisons, probably due to the problems that may be encountered while studying magnetofection in vivo.

The first problem of the precise magnetic guidance of complex magnetic nanostructures is the effect of applied magnetic fields. It is clear that an external magnetic field of higher intensity and gradients contributes to the faster accumulation of the magnetic nanosystems in a target region. On the other hand, an excessively strong magnetic force will result in too intense aggregation of nanoparticles, thus affecting their stability and causing potential cytotoxicity [43]. In turn, a nonlinear decrease in magnetic force with an increasing distance inevitably leads to a weaker response to the external magnetic field, which may become not strong enough to steer the magnetic nanoparticles in the blood flow deep in the body [82]. It would be interesting to study the transgene expression applied with an external magnetic field of stronger gradients, which could penetrate at a larger distance on an intact animal model. In addition, it is difficult, using the current approach, to eliminate completely gene expression in other organs, at least with the commonly used NdFeB magnets. This emphasizes the need for a more focused magnetic field of a higher gradient as, for example, in magnetic systems based on NdFeB micromagnets [83]. In particular, it is reported that the magnetic targeting of deeper situated tissues or of structures situated deeper within organs is weakened, and the enhanced permeability and retention effect, in this case, plays a greater role in biodistribution than the gradient magnetic field [51,84]. To overcome this problem, one of the approaches is to use field-enhancing elements as noted in [85,86,87,88], where vascular stents were made magnetic by nickel coating. If implanting these into a deeper situated vessel, these may influence the original magnetic field when magnetized by an external magnetic field and thus may generate strong field gradients deeper into the tissues. Another example of such an invasive technique is the implantation of ferromagnetic wires and exposure of the target area or the whole patient body to an external magnetic field. In this way, strong magnetic field gradients are produced locally and allow capture and concentration of circulating magnetic nanoparticles [89,90]. Alternatively, high gradient [91] or oscillating [92,93,94] magnetic fields can be used for standard magnetic nanoparticles. Another option is an employment of magnetic materials with higher specific magnetization than iron oxide, but this approach raises biocompatibility concerns. A comparison between magnetite and cobalt nanoparticles (the latter exhibiting a higher magnetization than that of magnetite) of identical sizes and coatings demonstrated similar transfection efficiencies but cobalt cytotoxicity was greater, and the nanoparticles tended to aggregate [38]. For a closer look at modern magnetic systems that allow precise magnetic guiding, we direct the reader to detailed reviews [43,95,96,97].

The second problem is that in order to further improve the binding of ligands to specific cells, it is necessary to introduce functional ligands such as galactose, folic acid, epithelial cell adhesion molecule and α-fetoprotein that can actively interact with the corresponding binding sites on the cell surfaces [48,55,98]. For example, it is claimed in [48] that conjugation of magnetic nanosystem with a vasculature-permeabilizing agent, such as histamine [99], VEGF [100], or serotonin [101], may further help such complex magnetic nanostructures to cross the endothelial barrier and deliver therapeutic genes to other cell types.

The third problem is that magnetic nanostructures during circulation in the bloodstream inevitably encounter difficulties associated with their interactions with blood components. For example, it is known that cationic nanoparticles attract opsonizing proteins. That causes rapid plasma clearance of the nanocarriers leading to short nanoparticle plasma half-life [102]. Therefore, it is necessary to study in detail the issues related to the factors that affect blood circulation [8,103], as well as elimination [104,105] of nanoparticles for successful applications of in vivo magnetofection, search for new hybrid delivering nanomaterials [106,107,108,109] or implementation of more efficient biochemical binding and targeting techniques [110,111,112,113,114,115,116]. However, we believe that the magnetofection technique can become a solid option for in vivo targeting combined with improved efficacy of gene delivery, potentially in combination with other physical techniques (electroporation, sonoporation) if appropriate magnetic fields can be generated and if an ideal coating of magnetic particles is found. Still, many questions and problems remain to be addressed before it becomes an efficient and optimized clinical tool for gene therapy.

## Figures and Tables

**Table 1:** Typical targets, nucleic acid types, magnetic nanoparticle compositions used in published research on magnetofection in different animals after systemic injections of magnetic carriers.

Target (Tissue/Organ)	Animals	Nucleic Acid Type ^a^	Magnetic Nanoparticle Composition ^b^	References
Subcutaneous tumor/heart	Mouse	shRNA	combiMAG + Lp2000	[44,45,46]
Subcutaneous tumor	Mouse	pDNA	Fe_3_O_4_@SiO_2_–COOH + PEI	[47]
Lungs/heart	Mouse	pDNA	MNBs (Miltenyi Biotec)-PEI	[48]
Right proximal region (subcutaneous tumor)	Mouse	siRNA	LipoMag	[10]
Right testis	Mouse	Antisense ODN	PolyMag	[49]
Liver	Rat	pDNA	MCL	[50]
Hind leg	Mouse	pDNA	PAAIO + CP	[51]
Striatum	Mouse	siRNA	Tf-PEG-PLL/MNP	[52]
Subcutaneous tumor/armpit	Mouse	siRNA	Fe_3_O_4_ + Chitosan	[53]
dorsal flank (subcutaneous tumor)	Mouse	pDNA	M-MSNs	[54]
Capsule of the liver lobe (transplanted tumor)	Mouse	pDNA	Gal-CMCS-Fe_3_O_4_-NPs	[55]
Left hepatic lobe	Mouse	siRNA	Gal-PEI-SPIO	[56]
Heart	Mouse	pDNA	MNB/PEI	[57]
Subcutaneous tumor	Mouse	pDNA	TSMCL	[58]
Vessels of the dorsal skin	Mouse	pDNA	MMB	[59]

^a^ pDNA = plasmid DNA, Antisense ODN = antisense oligonucleotide, siRNA = small interfering RNA, ^b^ combiMAG = commercial magnetofection reagent, Lp2000 = Lipofectamine^®^2000—commercial transfection reagent, PEI = polyethylenimine, MNB = magnetic nanobeads (MiltenyiBiotec, Auburn, CA, USA), LipoMag=commercial transfection kit, PolyMag (Chemicell, Berlin, Germany) = commercial iron oxide nanoparticles, MCL = magnetic cationic liposome, PAAIO = poly(acrylic acid)—bound superparamagnetic iron oxide, CP = polyethylenimine copolymer, Tf = transferrin, PEG = polyethylene glycol, PLL = poly-l-lysine, MNP = magnetic nanoparticle, M-MSNs = magnetic mesoporous silica nanoparticles, Gal = galactose, CMCS = carboxymethyl chitosan, NPs = nanoparticles, SPIO = superparamagnetic iron oxide, TSMCL = magnetic 1,2-dipalmitoyl-sn-glycero-3-phosphocholine (DPPC), 3b-[*N*-(*N*’,*N*’-dimethylaminoethane)-carbamoyl]cholesterol (DC-Cholesterol), dimethyldioctadecylammonium bromide (DOAB) and cholesterol liposomes at a molar ratio of 80:5:5:10, MMB = micromagnetobubbles.

## Data Availability

Not applicable.
